# A Randomized Trial Comparing Suprachoroidal and Intravitreal Injection of Triamcinolone Acetonide in Refractory Diabetic Macular Edema due to Epiretinal Membrane

**DOI:** 10.1155/2022/7947710

**Published:** 2022-01-21

**Authors:** Ahmed Abdelshafy Tabl, Tarek Tawfik Soliman, Mohamed Anany Elsayed, Marwa Abdelshafy Tabl

**Affiliations:** Department of Ophthalmology, Faculty of Medicine, Benha University, Farid Nada St., Banha 13511, Egypt

## Abstract

**Purpose:**

To compare the efficacy and safety of suprachoroidal and intravitreal injection of triamcinolone acetonide in pseudophakic patients with refractory diabetic macular edema (DME) due to epiretinal membrane (ERM). *Study Design*. This study is a randomized clinical trial (RCT). *Participants*. Twenty-three nonvitrectomized pseudophakic eyes of 23 subjects (9 M and 14 F with mean age: 54.8 years) with refractory DME due to ERM.

**Methods:**

The eyes were randomized to suprachoroidal triamcinolone acetonide injection 4 mg/0.1 ml (SCTA) (*n* = 13 eyes) or intravitreal triamcinolone acetonide 4 mg/0.1 ml (IVTA) (*n* = 10 eyes) and were evaluated at baseline and 1 and 3 months after injection to assess outcome measures. *Main Outcome Measures*. Changes in best-corrected visual acuity (BCVA) (primary outcome), central foveal thickness (CFT) by optical coherence tomography (OCT), and intraocular pressure (IOP) measurement (secondary).

**Results:**

Baseline median BCVA (logMAR) was 1.0 (range 0.8–1.0) in both groups, improved within the SCTA group to 0.8 on the 1^st^ and 3^rd^ months, while in the IVTA group, median BCVA changed to 0.8 and 0.9 on the 1^st^ and 3^rd^ months, respectively. No significant differences were noted between groups regarding BCVA at baseline (*P*=0.927), and 1^st^ (*P*=0.605) and 3^rd^ months (*P*=0.313). Regarding mean CFT, no significant differences were observed at baseline (*P*=0.353) and at the first month (*P*=0.214) between both groups, while at the third month, CFT was significantly higher in the IVTA group (385 um) than in the SCTA group (323 um) (*P*=0.028). Mean IOP was significantly higher in the IVTA group (15 mmHg) on 1^st^ month than in the SCTA group (12 mmHg) (*P*=0.011); after 3^rd^ month, IOP was significantly higher within the IVTA group (18 mmHg) than SCTA (14 mmHg) (*P*=0.028). No significant difference was noted between both groups at baseline IOP (*P*=0.435).

**Conclusions:**

Both SCTA and IVTA are effective in reduction of CFT and improvement of patients' visual acuity, but with a higher recurrence rate and rise in IOP after IVTA when compared to SCTA. Both treatments have temporary effects with the possibility of recurrence of DME and the need for retreatment.

## 1. Introduction

Diabetic macular edema (DME) is the most common ocular complication of diabetes that may cause serious vision problems. DME can occur at any stage of diabetic retinopathy (DR), although it is more likely to occur as the disease progresses [1]. Poor glucose control over a long period of time, very high blood pressure, and hyperlipidemia may increase risk for DME. Treatment of DME commonly includes argon laser photocoagulation, intravitreal injection of antivascular endothelial growth factor (VEGF) agents, or corticosteroids [2, 3]. Anti-VEGF agents as ranibizumab, bevacizumab, and aflibercept can achieve a significant improvement in visual acuity; however, repeated injections can induce serious side effects such as intraocular inflammation, infectious endophthalmitis, and even myocardial infarction or stroke. Moreover, anti-VEGF treatment could not be suitable therapy for all patients, especially those with poor response despite monthly injections [2]. The pathophysiology of DME has been well known to be multifactorial; in addition to VEGF, inflammation may be another feature of this disease [4, 5]. Refractory or persistent DME is defined as central foveal thickness (CFT) greater than 250 *μ*m measured by optical coherence tomography (OCT) with lack of visual improvement, despite monthly injections of anti-VEGF for at least 6 months [6].

The incidence of epiretinal membrane (ERM) formation in DME increases with various risk factors, such as old age, cataract surgery, and panretinal photocoagulation [7]. Previous studies concluded that T and B lymphocytes were more in ERM removed from the eyes with proliferative diabetic retinopathy (PDR), and their cell density reflected the severity of diabetic retinopathy [8]. It has been reported that secondary ERM has occurred after repeated intravitreal injections of anti-VEGF agents [9]. The treatment of refractory DME due to ERM is still controversial; on the other hand, anteroposterior tractions as vitreomacular traction (VMT) or proliferative preretinal membranes usually require pars-plana vitrectomy with or without peeling of internal limiting membrane (ILM) [7, 10].

Corticosteroids have both anti-inflammatory and angiostatic effects, they have been used in the form of intravitreal injections or implants as a second-line therapy in the treatment of DME due to their side effects as cataract progression and ocular hypertension, in refractory DME due to ERM corticosteroids therapy is considered the first line of treatment due to their effect on various inflammatory mediators [11].

Dexamethasone implant (DI) is a common form of steroids used in cases of refractory DME due to ERM; a recent study conducted by Erden et al. concluded that the presence of ERM limited efficacy of DI in reducing CFT and the recurrence of DME occurred at the same baseline levels after 6 months of follow-up with a significant increase in intraocular pressure (IOP) [7, 11, 12].

Suprachoroidal injection is a novel therapy for intraocular drug delivery with higher concentration towards posterior segment of the eye compared to intravitreal route [13]. Hulk trial recently concluded that suprachoroidal triamcinolone acetonide injection (SCTA) is effective and safe in treating the eyes with DME achieving anatomical improvement with low incidence of adverse effects, and repeated SCTA injections were well tolerated [14].

In this study, our objective was to compare efficacy and safety of suprachoroidal and intravitreal injection of triamcinolone acetonide in the pseudophakic eyes with refractory diabetic macular edema due to epiretinal membrane with no vitreomacular traction.

## 2. Methods

In this randomized clinical trial, twenty-three pseudophakic eyes diagnosed as refractory DME due to ERM were enrolled in the retina clinic at Ophthalmology Department, Benha University Hospital, Egypt, and Ebsar Eye Center, Benha, Egypt, from February 2020 to April 2021.

The findings of the ophthalmological examination were included. Best-corrected visual acuity (BCVA) was measured with the Snellen chart and then converted to logMAR. IOP was measured by Goldmann applanation tonometry (AT 900, Haag-Streit Inc., the USA). The diagnosis of diabetic retinopathy and macular edema was made with slit-lamp using auxiliary +90D lens and fundus fluorescein angiography, central foveal thickness, and presence of ERM by OCT (Optovue, Fremont, CA 94538, the USA).

The patients were randomly assigned to one of two groups. The suprachoroidal triamcinolone acetonide group (SCTA) group (number of eyes: 13) received a single injection with a dose of 4 mg/0.1 ml and the intravitreal triamcinolone acetonide (IVTA) group (number of eyes: 10) received a single injection with a dose of 4 mg/0.1 ml. Patients were followed one and three months after injection to assess changes in BCVA, CFT, and IOP from baseline measures.

Written informed consent was obtained from all patients included in this study after discussing the surgical procedures to be performed and possible side effects of it.

This study was approved by the Faculty of Medicine, Benha University, and was carried out in compliance with principles of the Declaration of Helsinki.

Our inclusion criteria were as follows: type II diabetes mellitus, centrally involving DME with CFT greater than 300 *µ*m detected by OCT with posterior vitreous detachment and no anteroposterior vitreomacular traction, pseudophakic nonvitrectomized eyes with refractory DME due to ERM, and had no treatment for DME in the last three months at the time of recruitment. Our exclusion criteria were as follows: preexisting retinal disease other than diabetic retinopathy that may affect final visual outcome, ischemic diabetic macular edema detected by fundus fluorescein angiography, epiretinal proliferation that causes lamellar macular hole or contracted ERM detected by OCT, the glaucomatous eyes in which IOP ≥21 mmHg measured by Goldmann applanation tonometry and/or asymmetric vertical cup disc ratio or glaucoma suspect patients, central corneal opacity, vitreous hemorrhage, uncooperative patients or patients with poor fixation, and patients on systemic medications that could affect the macular thickness and uncontrolled systemic diseases.

Surgical techniques were performed in the operating room under complete sterile conditions by the same surgeon. The surgical preparations were the same for both injection techniques, topical anesthetic eye drop (benoxinate hydrochloride 0.4%, Benox, Epico, Egypt) was instilled into the conjunctival sac, 10% periocular povidone-iodine was applied on the skin, and 5% povidone-iodine was applied on the conjunctiva after insertion of the sterile speculum and eye draping. We used 4 mg/0.1 ml of triamcinolone acetonide (epirelefan 40 mg/1 ml suspension, EPICO, Egypt) using the nonfiltering sedimentation technique for the purification of triamcinolone acetonide suspension.

### 2.1. Suprachoroidal Injection Technique

Suprachoroidal injection was done using custom made needle preparation to expose only 1 mm of the 27-gauge needle to enter the suprachoroidal space; we used the silicon tube with the sterile 22-gauge blue IV cannula (Rays Hemoflon, Italy) to use it as a guard for the 27-gauge needle (Rays 1 ml insulin syringe and 27 g needle, 13 mm Insu/Light, Italy); after cutting the silicon tube from its base, we insert the 27-gauge needle inside; then, we cut the silicon tube by Westcott scissor to expose only 1 mm of the 27-gauge needle bevel using a sterile caliper to measure the desired length. Surgeon aspirates 0.1 ml of triamcinolone suspension into a sterile 27-gauge syringe and then inserts the custom 27-gauge suprachoroidal needle we prepared. Paracentesis was created; then, suprachoroidal injection was done 3.5 mm posterior to the limbus in the inferotemporal quadrant.

### 2.2. Intravitreal Injection

Intravitreal injection was placed 3.5 mm posterior to the limbus after paracentesis using a standard 27-gauge needle (1 ml insulin syringe and 27 g needle).

### 2.3. Postoperative Management

Fourth-generation fluoroquinolone eye drop is used 4 times per day for one week for all patients (moxifloxacin 0.5%, Vigamox, Alcon Lab Ind.), and patients were instructed for follow-up schedule 2 days, one week, and one and three months after the procedure. BCVA, CFT, and IOP were measured for all patients one and three months after surgery for each group.

### 2.4. Statistical Analysis

Data management and statistical analysis were done using SPSS version 25 (IBM, Armonk, New York, United States). Quantitative data were assessed for normality using the Shapiro–Wilk test and direct data visualization methods. According to normality testing, numerical data were summarized as means and standard deviations or medians and ranges. Categorical data were summarized as numbers and percentages. Quantitative data were compared between study groups using the independent *t*-test or Mann–Whitney *U* test for normally and nonnormally distributed numerical variables, respectively. Categorical data were compared using the chi-square test. Within-group comparisons were made using repeated-measures ANOA or Friendman's test for normally and nonnormally distributed numerical variables. All statistical tests were two-sided. *P* values less than 0.05 were considered significant. Kaplan–Meier was done to estimate time to recurrence in both groups.

## 3. Results

### 3.1. Demographics

Twenty-three eyes of 23 patients were included in this study (9 males and 14 females with mean age of 54.8 years).

No significant differences were observed between the two groups regarding age (*P* value = 0.808) and gender (*P* value = 0.431) (Table 1).

Regarding BCVA, no significant differences were noted between both groups at baseline BCVA (*P* value = 0.927), 1^st^ month (*P* value = 0.605), and 3^rd^ month (*P* value = 0.313) (Table 2).

**Figure 1:**
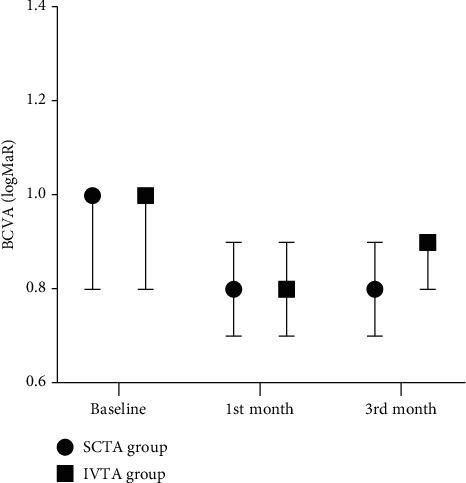
BCVA in both groups at baseline, one month, and three months.

Regarding the SCTA group, BCVA showed an overall significant improvement after 3^rd^ month (*P* value <0.001). Post hoc analysis revealed that it was significantly higher at baseline (1 logMAR) than 1^st^ and 3^rd^ months (0.8 logMAR for each). Furthermore, the IVTA group showed a significant improvement in BCVA at 3^rd^ month (*P* value was = 0.003). Post hoc analysis showed that it was significantly higher at baseline (1.0 logMAR) than at the first and 3rd months (0.8 and 0.9 logMAR, respectively) (Figure 1).

The central foveal thickness at three months was significantly higher in the IVTA group (385 um) than in the SCTA group (323 um); the *P* value was 0.028. No significant differences were noted at baseline (*P* value = 0.353) and at 1^st^ month (*P* value = 0.214) between both groups.

**Figure 2:**
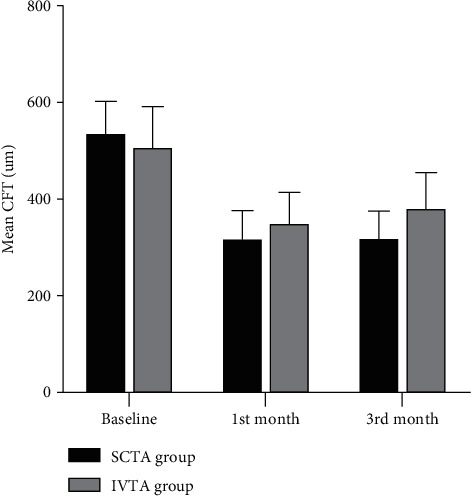
Central foveal thickness in both groups at baseline, 1, and 3 months.

Within the SCTA group, CFT showed an overall significant reduction (*P* value <0.001). Post hoc analysis revealed that it was significantly higher at baseline (541 um) than at the 1^st^ and 3^rd^ months (322 um and 323 um, respectively). Also, within the IVTA group, CFT showed an overall significant improvement (*P* value was <0.001). Post hoc analysis revealed that it was significantly higher at baseline (512 um) than at the 1^st^ and 3^rd^ months (354 um and 385 um, respectively) (Table 3 and Figure 2).

**Figure 3:**
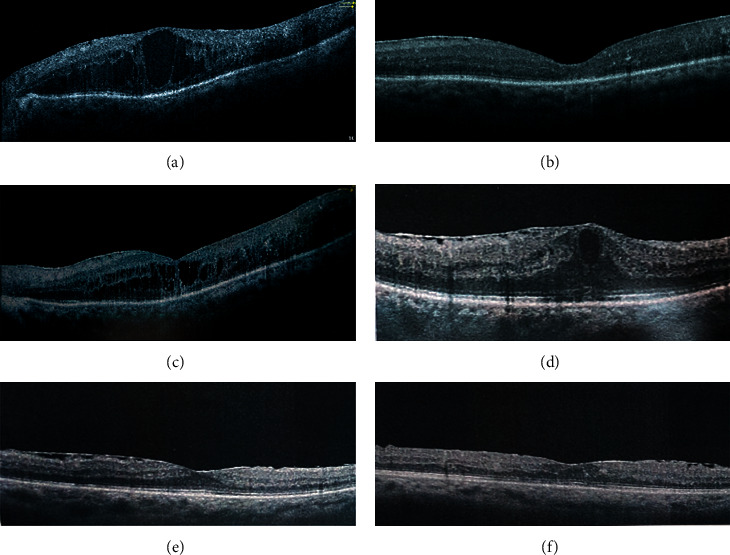
Example of CFT changes measured by OCT in both groups at baseline, 1, and 3 months. IVTA group (a)–(c). SCTA group (d)–(f).

Figure 3 shows an example of the changes in CFT measured by OCT in both groups. In the IVTA group, (a) baseline CFT was 643 um, (b) one month after injection, CFT was reduced to 226 um, and (c) 3^rd^ month CFT was 331 um, while in the SCTA group (d) baseline CFT was 658 um, (e) on 1^st^ month, CFT was reduced to 275 um, and (f) on 3^rd^ month CFT was 302 um.

**Figure 4:**
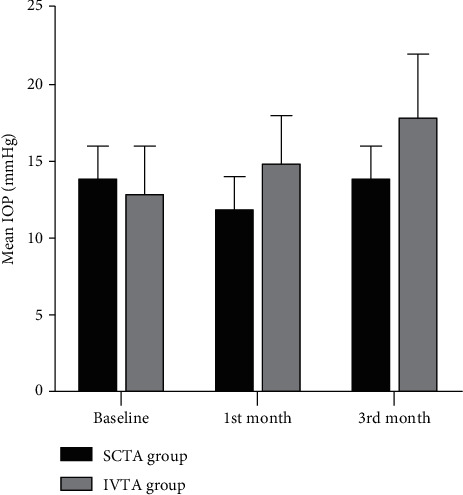
IOP in both groups at baseline, one month, and three months.

Regarding, IOP at one month was significantly higher in the IVTA group (15 mmHg) than in the SCTA group (12 mmHg). *P* value = 0.011. Furthermore, in the third month, it was significantly higher in IVTA (18 mmHg) than in the SCTA group (14 mmHg). *P* value was 0.028. No significant difference was noted between both groups at baseline (*P* value = 0.435) (Table 4 and Figure 4).

### 3.2. Time to Recurrence

**Figure 5:**
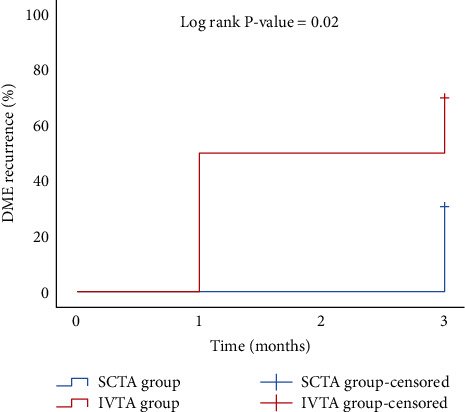
Kaplan–Meier analysis for time to DME recurrence in both groups.

Kaplan–Meier analysis was done to estimate time to DME recurrence in both groups, and a significant difference was observed between the two curves (log-rank *P* value = 0.02). At one month, the recurrence rate was 50% in the IVTA group and 0% in the SCTA group. After 3 months, the recurrence rate was 70% in the IVTA group and 30.8% in the SCTA group (Figure 5).

## 4. Discussion

This randomized clinical trial compared the efficacy and safety of SCTA and IVTA injection in the pseudophakic eyes with refractory DME due to ERM without VMT, and we found significant short-term improvement in BCVA and CFT in all groups after one month of injection with higher incidence of DME recurrence among patients treated with IVTA at 3^rd^ month follow-up.

Medical effective and safe therapy for refractory DME due to ERM is still controversial. Previous studies reported poor outcome for anti-VEGF agents in DME with concurrent ERM, and this was explained as ERM has a barrier effect due to its inflammatory origin [15, 16]. DI is an effective treatment for persistent DME, but it has a short-term efficacy in cases with coexisting ERM [16]; in a study by Cakir et al., 22 eyes with DME due to ERM were injected with DI and had a gradual decrease in BCVA and recurrence in retinal edema 3 months after surgery [17]. Erden and his colleagues reported a significant rise in IOP after DI in 49 eyes after single and multiple DI during 3 months of follow-up [7].

Intravitreal corticosteroids use in DME is limited in phakic and glaucomatous patients due to steroid-related complications. In a study conducted by Brasil et al., IVTA injection was done in 73 eyes with DME, ERM affected the baseline and final outcome of BCVA and CFT, and cataract progression occurred in 38.7% and increased IOP more than 20 mmHg in 8.2% that was controlled by medical treatment [18]; in our study, 30% of the IVTA group had IOP over 20 mmHg and were also controlled medically.

Recently, SCTA has been used as a minimally invasive therapy in cases of DME achieving a higher drug concentration targeting the retina and choroid with lesser effects on the anterior segment of the eye [19], the Hulk trial investigated efficacy of SCTA in 10 patients with persistent DME [14], the mean baseline CFT in the previously treated arm of the HULK trial was 473 um; whereas, in this study, it was 541.2 ± 64 um. After 6 months, mean CFT in HULK was reduced to 369 um; whereas, in this study, mean CFT in the SCTA group after three months was reduced to 323 ± 54 um.

An increase in IOP was observed in 10% of patients in the Hulk trial [14], and these results are consistent with ours within the SCTA group; only one eye (7.6%) had elevated IOP up to 18 mmHg at the third month follow-up and were treated medically; in the Tanzanite study [20], a higher incidence of ocular hypertension was observed (17.3%), but this may be related to the combination of intravitreal aflibercept with SCTA and preexisting glaucoma in cases of retinal vein occlusion.

Regarding other ocular adverse effects in both groups, no endophthalmitis and suprachoroidal hemorrhage, localized subconjunctival hemorrhage occurred in one eye in the SCTA group (7.6%) and two eyes in the IVTA group (20%); we faced no cases with inadvertent intravitreal injection among SCTA; whereas, the Hulk trial reported one case of inadvertent Intravitreal injection [14].

Both SCTA and IVTA have a short-term effect in improving BCVA in refractory DME due to ERM with the possibility of recurrence; however, SCTA has a lower incidence of ocular hypertension and recurrence of DME; this study has several limitations, such as relatively small sample size and short-term follow-up, and more studies are needed to investigate the long-term efficacy of SCTA in cases of DME with ERM and the possibility of repeated injections of SCTA injections to achieve a long-term anatomical and functional improvement with the delay of surgical removal of ERM in these cases.

## Figures and Tables

**Table 1:** Demographic characteristics in both groups.

Demographics		SCTA group (*n* = 13 eyes)	IVTA group (*n* = 10 eyes)	*P* value
Age (years)	Mean ± SD	55 ± 3	55 ± 5	0.808
Gender	Male, *n* (%)	6 (46.2)	3 (30.0)	0.431
	Female, *n* (%)	7 (53.8)	7 (70.0)	

The independent *t*-test was used for age. The chi-square test was used for gender. SCTA, suprachoroidal triamcinolone acetonide; IVTA, intravitreal triamcinolone acetonide.

**Table 2:** BCVA in both groups at baseline, one month, and three months.

BCVA (logMAR)		SCTA group (*n* = 13 eyes)	IVTA group (*n* = 10 eyes)	*P* value
Baseline	Median (range)	1 (0.8–1)^a^	1 (0.8–1.0)^a^	0.927
1^st^ month	Median (range)	0.8 (0.7–0.9)^b^	0.8 (0.7–0.9)^b^	0.605
3^rd^ month	Median (range)	0.8 (0.6–0.9)^b^	0.9 (0.8–0.9)^a,b^	0.313
*P* value		<0.001	0.003	

The Mann–Whitney *U* test was used between groups. Within-group comparisons were done using Friedman's test. Post hoc analysis was done using Bonferroni's method, and different letters indicate a significant pair. BCVA, best-corrected visual acuity; SCTA, suprachoroidal triamcinolone acetonide; IVTA, intravitreal triamcinolone acetonide.

**Table 3:** Central foveal thickness in both groups at baseline, 1, and 3 months.

CFT (um)		SCTA group (*n* = 13 eyes)	IVTA group (*n* = 10 eyes)	*P* value
Baseline	Mean ± SD	541 ± 64^a^	512 ± 82^a^	0.353
1^st^ month	Mean ± SD	322 ± 56^b^	354 ± 62^b^	0.214
3^rd^ month	Mean ± SD	323 ± 54^b^	385 ± 72^b^	0.028
*P* value		<0.001	<0.001	

The independent *t*-test was used between the groups. Within-group comparisons were done using repeated-measures ANOVA. Post hoc analysis was done using Bonferroni's method, and different letters indicate significant pair. CFT, central foveal thickness; SCTA, suprachoroidal triamcinolone acetonide; IVTA, intravitreal triamcinolone acetonide.

**Table 4:** IOP in both groups at baseline, one month, and three months.

IOP (mmHg)		SCTA group (*n* = 13 eyes)	IVTA group (*n* = 10 eyes)	*P* value
Baseline	Mean ± SD	14 ± 2^a^	13 ± 3^a^	0.435
1^st^ month	Mean ± SD	12 ± 2^b^	15 ± 3^b^	0.011
3^rd^ month	Mean ± SD	14 ± 2^a^	18 ± 4^c^	0.028
*P* value		<0.001	<0.001	

The independent *t*-test was used between the groups. Within-group comparisons were done using repeated measures ANOVA. Post hoc analysis was done using Bonferroni's method, and different letters indicate significant pair. IOP, intraocular pressure; SCTA, suprachoroidal triamcinolone acetonide; IVTA, intravitreal triamcinolone acetonide.

## Data Availability

The data of patients used to support the results of this study are limited by the Research Ethics Committee of the Faculty of medicine, Benha University. Data are available to researchers who meet the criteria for accessing confidential data from the corresponding author upon request.

## Abbreviations

BCVA: Best-corrected visual acuity
CFT: Central foveal thickness
DI: Dexamethasone implant
ILM: Internal limiting membrane
IOP: Intraocular pressure
IVTA: Intravitreal triamcinolone acetonide
LogMAR: Logarithm of minimum angle of resolution
OCT: Optical coherence tomography
PDR: Proliferative diabetic retinopathy
SCTA: Suprachoroidal triamcinolone acetonide
VEGF: Vascular endothelial growth factors
VMT: Vitreomacular traction.

## Additional Points

Human subjects were included in this study.
